# Myocardial contractility in the echo lab: molecular, cellular and pathophysiological basis

**DOI:** 10.1186/1476-7120-3-27

**Published:** 2005-09-08

**Authors:** Tonino Bombardini

**Affiliations:** 1 Department of Echocardiography, Institute of Clinical Physiology, National Council of Research, Pisa, Italy

## Abstract

In the standard accepted concept, contractility is the intrinsic ability of heart muscle to generate force and to shorten, independently of changes in the preload or afterload with fixed heart rates. At molecular level the crux of the contractile process lies in the changing concentrations of Ca^2+ ^ions in the myocardial cytosol. Ca^2+ ^ions enter through the calcium channel that opens in response to the wave of depolarization that travels along the sarcolemma. These Ca^2+ ^ions "trigger" the release of more calcium from the sarcoplasmic reticulum (SR) and thereby initiate a contraction-relaxation cycle.

In the past, several attempts were made to transfer the pure physiological concept of contractility, expressed in the isolated myocardial fiber by the maximal velocity of contraction of unloaded muscle fiber (Vmax), to the in vivo beating heart. Suga and Sagawa achieved this aim by measuring pressure/volume loops in the intact heart: during a positive inotropic intervention, the pressure volume loop reflects a smaller end-systolic volume and a higher end-systolic pressure, so that the slope of the pressure volume relationship moves upward and to the left. The pressure volume relationship is the most reliable index for assessing myocardial contractility in the intact circulation and is almost insensitive to changes in preload and after load. This is widely used in animal studies and occasionally clinically. The limit of the pressure volume relationship is that it fails to take into account the frequency-dependent regulation of contractility: the frequency-dependent control of transmembrane Ca^2+ ^entry via voltage-gated Ca2^+ ^channels provides cardiac cells with a highly sophisticated short-term system for the regulation of intracellular Ca^2+ ^homeostasis. An increased stimulation rate increases the force of contraction: the explanation is repetitive Ca^2+ ^entry with each depolarization and, hence, an accumulation of cytosolic calcium. As the heart fails, there is a change in the gene expression from the normal adult pattern to that of fetal life with an inversion of the normal positive slope of the force-frequency relation: systolic calcium release and diastolic calcium reuptake process is lowered at the basal state and, instead of accelerating for increasing heart rates, slows down. Since the force-frequency relation uncovers initial alteration of contractility, as an intermediate step between normal and abnormal contractility at rest, a practical index to measure it is mandatory.

Measuring end-systolic elastance for increasing heart rates is impractical: increasing heart rates with atrial pacing has to be adjunct to the left ventricular conductance catheter, to the left ventricular pressure catheter, to the vena cava balloon, and to afterload changes. Furthermore, a noninvasive index is needed. Noninvasive measurement of the pressure/volume ratio for increasing heart rates during stress in the echo lab could be the practical answer to this new clinical demand in the current years of a dramatic increase in the number of heart failure patients.

## Calcium channels: evolutional aspects

When ~3,500,000,000 years ago prokaryotes appeared, the selection of an intracellular messenger preceded the appearance of ionic channels of enveloping lipid membranes.

Calcium had conformational and stechiometric advantages to be chosen as an intracellular messenger (=good messenger and modulator of intracellular processes), due to its high coordination number and irregular coordination geometry. If calcium was the first intracellular messenger, ionic channels for calcium had to appear for the cells to maintain constant intracellular calcium concentrations [1]. Ionic channels, other than function of intracellular ionic concentration surveyors, became to have the first function of reactive capability to outer stimulus, changing abruptly their functions. The primitive Ca^2+ ^channels were activated by mechanical stimuli, present but slow and low efficient in reactions. But faster reactions are essential for survival.

When ~1,500,000,000 years ago the hydrosphere became aerobic and the primitive unicellular organisms developed mechanisms of energy production, eukaryotes developed active transport and voltage-gated channels as a result of selection for faster signaling [1,2].

When in this new evolutional cell a mechanical stimulus involving a portion of the membrane hits the cell, a depolarization produced by the mechanic-sensitive Ca^2+ ^channels subsequently extends the effect to the entire cell by voltage gated Ca^2+ ^channels.

### Toward the Na channel: high-speed signaling required by multicellularity

With the appearance of multicellularity, even faster signaling had to appear for life competition. As a possible solution to this new requirement, increase in density of Ca^2+ ^channels would increase the speed of the depolarizing wave, but would have compromised the role of Ca^2+ ^as modulator of intracellular function.

The appearance of Na^+ ^channels, capable of carrying greater ionic fluxes without interfering with intracellular processes, would be evolutionarily favorable. And, in fact, this model was the one chosen by evolution in nerve cells: earlier selection of Na^+ ^channels to sustain potential changes [1]. In fact, also for a maximum concentration on the cell membrane of channels, Ca^2+ ^channels mediated maximum velocity is equal to 0.10 m/sec. For Na^+ ^channels mediated max. velocity is 3 m/sec.

### Muscle fibers: a particular evolutionary aspect

In primitive muscle fibers all the activating Ca^2+ ^ions for contraction came from outside the cell.

High density of Ca^2+ ^channels would increase the speed of the depolarizing wave and at the same time would speed up the activation of the contractile machinery.

But with the evolutionary appearance of intracellular calcium stores (sarcoplasmic reticulum) in supplying Ca^2+ ^ions for contraction, muscle fibers acquired the capability of stronger contraction with less energy consumption. As for force of contraction, also signaling speed was a problem in muscle cells: but in these cells less pronounced advantages are provided by an exclusively Na^+^-channels-based membrane potential changes. The simultaneous presence of fast Na^+ ^channels for conduction function and slow Ca^2+ ^channels for beginning the contraction mechanism through inward calcium flow was maintained [3].

At the present evolutionary state, once the impulse has formed in the sinus node, it spreads very rapidly throughout the atrium to reach the atrioventricular (AV) node and ventricles. In atrial tissue, the pattern of the action potential is dominated by a fast sodium channel. The action potential duration of atrial tissue is short when compared to that of ventricles, and the inward flow of calcium ions is less, with a lower force of contraction developed in the atria and lower activity of L-type calcium channels. Conduction of the wave of depolarization is rapid through conduction tissues where the action potential goes through fast sodium channel activity, whereas conduction is lower through the ventricular myocardium, where there is chiefly calcium channel activity with a slower rate of depolarization.

### High frequency-induced upregulation of human cardiac calcium currents: the final evolution

Ultimately developed, the frequency-dependent control of transmembrane Ca^2+ ^entry via voltage-gated Ca^2+ ^channels provides mammalian cardiac cells with a highly sophisticated short-term system for regulation of intracellular Ca^2+ ^homeostasis.

**Figure 1:**
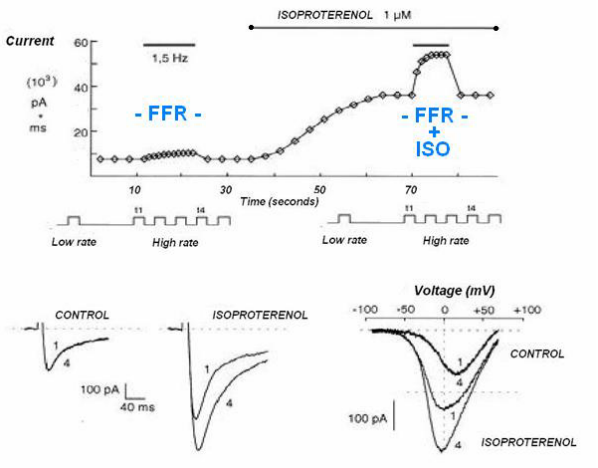
High frequency-induced upregulation of human cardiac calcium currents in isolated cardiomyocytes. Up regulation of Ca^2+ ^entry through Ca^2+ ^channels by high rates of beating is involved in the frequency-dependent regulation of contractility: for each increasing heart rate the steady state is reached rapidly (within few seconds, on the left : FFR). Beta-adrenergic receptor stimulation produces an important enhancement of the force-frequency relation on myocardial contractility: β-adrenergic stimulation, by means of cyclic adenosine monophosphate, promotes phosphorylation and the opening probability of the Ca^2+ ^channel. The effect of increasing contractility by increasing heart rate ("pure" Bowditch treppe) is intrinsic to myocardium and takes few seconds to occur, while the β-adrenergic amplification of the force-frequency relation takes longer, i.e. 30–40 seconds, the time it takes for β-receptor activation and cAMP synthesis (on the right: FFR + ISO). (Modified from: Piot C, Lemaire S, Albat B, et al. High frequency-induced upregulation of human cardiac calcium currents. Circ 1996; 93:120–8)

Up-regulation of Ca^2+ ^entry through Ca^2+ ^channels by high rates of beating (HFIUR of ICa) is involved in the frequency-dependent regulation of contractility: this process is crucial in adaptation to exercise and stress [4,5]. This regulation is rapid, (the steady state is reached rapidly within few seconds for each heart rate level), intrinsic to the myocardium cell, with no necessity to be driven from neuronal or hormonal controls (Fig. 1).

## Contractility

### Molecular aspects: calcium ion fluxes in cardiac contraction-relaxation cycle

The crux of the contractile process lies in the changing concentrations of Ca^2+ ^ions in the myocardial cytosol.

Crucial features are [6] entry of Ca^2+ ^ions through the voltage-sensitive L-type Ca^2+ ^channels, acting as a trigger for the release of Ca^2+ ^ions from the sarcoplasmic reticulum (SR).

**Figure 2:**
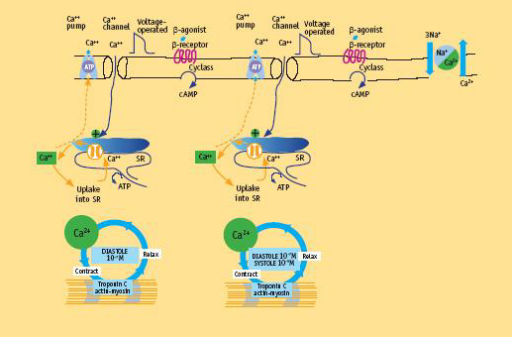
Molecular basis of contractility in normal heart. Crucial features are entry of Ca^2+ ^ions through the voltage-sensitive L-type Ca^2+ ^channels in response to the wave of depolarization, acting as a trigger for the release of Ca^2+ ^ions from the sarcoplasmic reticulum (SR). The crux of the contractile process lies in the changing concentrations of Ca^2+ ^ions in the myocardial cytosol. The varying actin-myosin overlap is shown for systole, when calcium ions arrive, and diastole, when calcium ions leave. At the end of systole, calcium stops interaction with troponin C and calcium ions are taken up into the SR by the activity of the pump called SERCA. Calcium taken up into the SR by the calcium uptake pump is stored within the SR before further release. The small amount of calcium that has entered the cell leaves predominantly through a Na^+^/Ca^2+ ^exchanger. (Modified from Opie LH. Normal and abnormal cardiac function. Chapter 14, page 443. In Braunwald Zipes Libby Heart disease, 6th edition, W. B Saunders Company, 2001)

Relatively small amounts of calcium ions actually enter and leave the cell during each cardiac cycle, whereas much larger amounts move in and out of the sarcoplasmic reticulum. Calcium-induced calcium release explains most of the current available data. This process elevates by about tenfold the concentration of calcium ions in the cytosol. The result is the increasing interaction of calcium ions with troponin C to trigger the contractile proteins [6] (Fig. 2).

#### Left ventricular contraction

Left ventricular pressure starts to builds up when the arrival of calcium ions at the contractile proteins starts to trigger actin-myosine interaction. The thin actin filament interacts with the myosin head when Ca^2+ ^ions arrive at troponin C (TnC). As more and more myofibers enter the contracted state, pressure development in the left ventricle proceeds. The interaction of actin and myosine increases, and cross-bridge cycling augments. As long as enough calcium ions are bound to troponin C, many repetitive cycles of this nature occur. The enhanced force development in response to a greater calcium ion concentration is due to recruitment of additional cross bridges.

When calcium ions depart from their binding sites on troponin C, cross-bridge cycling cannot occur and the diastolic phase of the cardiac cycle sets in.

#### Left ventricular relaxation

At the end of systole, calcium stops interacting with troponin C and calcium ions are taken up into the SR by the activity of the SERCA (sarcoplasmatic reticulum Ca^2+ ^ATPase) pump that constitutes nearly 90% of the protein component of the SR. Calcium taken up into the SR by the calcium uptake pump is stored within the SR before further release. To balance the small quantity of calcium ions entering the heart cell with each depolarization, a similar quantity must leave the cell. First, calcium can be exchanged for sodium ions entering by the Na^+^/Ca^2+ ^exchange and, second, an ATP-consuming sarcolemmal calcium pump can transfer calcium into this extra cellular space against a concentration gradient.

As the cytosolic calcium ion concentration starts to decline because of the uptake of calcium into the SR under the influence of activated phospholamban, more and more myofibers enter the state of relaxation (Fig. 2).

#### Preload and after load

The preload is the load present before contraction has started, at the end of diastole. When the preload increases, the left ventricle distends during diastole, and the stroke volume rises according to Starling's law [7]. The proposed explanation for the Starling effect, whereby a greater end-diastolic fiber length develops a greater force, is explained by an interaction between sarcomere length and calcium ions (length sensitization of the sarcomere): 1) increase in end-diastolic fiber length at any given free Ca^2+ ^concentration would increase force by a small amount on the basis of the change in filament overlap; 2) when the fiber is stretched and the sarcomere length increases, for any given number of Ca^2+ ^ions binding to TnC, there is greater force development. Length sensitization of the sarcomere explains how the sarcomere can "upgrade itself" to a higher force-length curve [6].

The afterload is the systolic load on the left ventricle after it has started to contract.

Increased afterload means that an increased intraventricular pressure has to be generated first to open the aortic valve and then during the ejection phase [8].

In the nonfailing heart, the left ventricle can overcome any physiological acute increase in load [6].

### Contractility: how can it be defined?

"Contractility is the inherent capacity of the myocardium to contract independently of changes in the preload or afterload. Whatever the problems of measuring it, contractility remains an essential corner concept to separate the effects of a primary change in loading conditions from an intrinsic change in the force of contraction [6]". It is a basic property of cardiac muscle and is strictly linked to the activation quantity of actin myosin transverse bridges in the myocardial fibers, and to the velocity of cross-bridge activation at the systole onset [6,9]. Cytosolic calcium level is the determinant of:

- The myocardial fiber number involved in the contraction process.

- The maximal velocity of myocardial fibers shortening.

Increased contractility, is reflected in higher myocardial fiber shortening velocity, with a more highly developed tension peak and a steeper pressure rise, when preload, afterload, and heart rate are constant: in the cytosol calcium release is more and faster from SR with a higher cytosol calcium concentration in systole: more troponin is activated from higher levels of calcium with more acitn-myosin cross-bridges in the time unit, and ultimately myocardial fiber contraction is more and faster.

Decreased contractility is reflected in lower myocardial fiber shortening velocity, with a lower tension peak and a blunted pressure rise, when preload, after load, and heart rate are constant: in the cytosol calcium release is less and slower from SR with a lower cytosol calcium concentration in systole: less troponin is activated from lower levels of calcium with less actin-myosin cross-bridges in time unit, and ultimately myocardial fiber contraction is less and slower.

### The isolated myocardial fiber: idealized contractility in the physio lab

Contractility expressed in the isolated myocardial fiber is the maximal velocity of contraction of unloaded muscle fiber (Vmax). This value is defined as the maximal velocity of contraction, when there is no load on the isolated muscle. This strictly preload and after load independent index, fulfills the theoretical requirements for contractility quantification and greatly contributes to this research field [9,10]. Nevertheless, this model is not usable in in-vivo conditions.

### The in vivo, beating heart: how to measure contractility

In the past attempts were made to transfer the purely physiological concept of contractility expressed in the isolated myocardial fiber by the maximal velocity of contraction of unloaded muscle fiber (Vmax), to the in vivo beating heart. Suga and Sagawa achieved this aim by measuring pressure/volume loops in the intact heart: during a positive inotropic intervention, the pressure volume loop reflects a smaller end systolic volume and a higher end-systolic pressure, so that the slope of the pressure volume relationship (Ees) moves upward and to the left [11,12]. Ees is the most reliable index for assessing (standard) myocardial contractility at rest in the intact circulation and is almost insensitive to changes in preload, and after load.

This is widely used in animal studies and occasionally clinically [6].

**Figure 3:**
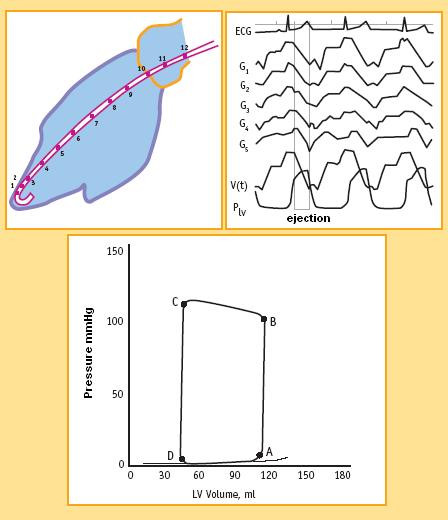
Pressure-volume loops in the cath lab. A conductance catheter is used to measure pressure-volume loops in humans. The time landmarks during the cardiac cycle include the following: B, aortic valve opening and the beginning of ejection; C, aortic valve closure; D, mitral valve opening; and A, end-diastole. During diastole (D-A tract) LV filling occurs, with a low end-diastolic LV pressure increase in the normal heart. During isovolumic contraction, or pre-ejection systole, (A-B tract) LV volume is unchanged but LV pressure rises to point B when it equals aortic pressure, and the aortic valve opens: isotonic systole, or systolic ejection phase (B-C tract), starts. When LV systolic emptying ends (C point), the aortic valve closes, and isovolumic diastolic relaxation starts. (C-D tract). Smaller end-systolic volume and higher end-systolic pressure are typical markers of higher contractility. Counter-directional changes identify compromised contractility. Focusing on end-systolic volume and on end-systolic pressure it immediately appears that the upper left corner of the pressure volume loop (C point) quantifies both measures.

A now-time conductance catheter is used for human studies [13]. The method is highly correct, but invasive, complex, and technically demanding. (Fig. 3)

Focusing on cytosol calcium concentrations along the pressure-volume loop, (Fig 3) in diastole (D-A tract) cytosolic calcium is reuptake from cytoplasm and stored in the SR [6]. At the A end-diastolic point, the end-diastolic volume (or maximal myocardial fiber length) predicts contractile-proteins calcium-sensitivity of the upcoming systole according to the Starling's law [7]. The velocity of the pressure development in the isovolumic systole (A-B tract), and the ejection force in the isotonic systole (B-C tract) are both strictly linked to the contractile state. When LV systolic emptying ends (C point), the aortic valve closes, and isovolumic diastolic relaxation starts. (C-D tract).

More highly developed systemic pressure simultaneously with lower end-systolic volume is typical of higher contractility. Counter-directional changes identify compromised contractility.

**Figure 4:**
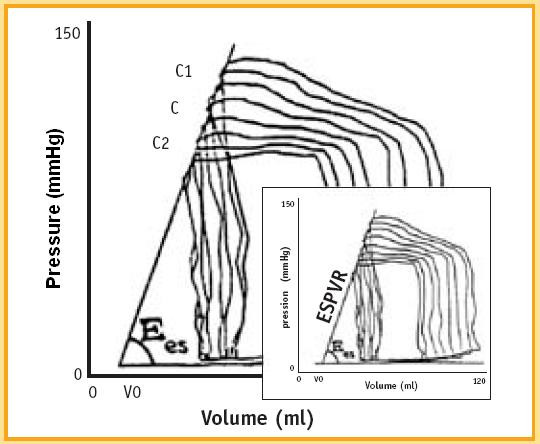
The end-systolic pressure-volume relationship (ESPVR). Suga and Sagawa were the first to use simultaneous LV pressure-volume measurements. These Authors, searching for a preload and afterload independent contractility index, measured pressure-volume loops during sudden preload and afterload changes. The upper left corners of the loops (C, C1, C2, C... points) define the LV end-systolic pressure-volume relation (ESPVR). ESPVR predicts the end-systolic volume in a heart with constant contractility when end-systolic pressure changes, and ultimately predicts the left ventricle ability to empty for different afterload values. The slope of the ESPVR line is the end-systolic elastance (Ees). In the clinical setting it is difficult to generate the end-systolic pressure-volume relationship (ESPVR) free of changes in reflex-mediated variations in contractility. It also requires a means to measure pressure and volume accurately and simultaneously.

If end-systolic volume is measured for different end-systolic pressure values, sequential end systolic pressure/volume values can be recorded (C, C1, C2, C... points). The upper left corners (C, C1, C2, and C... points) of the loops define the LV end-systolic pressure-volume relation (ESPVR). (Fig. 4) The ESPVR predicts in a heart with constant contractility the end-systolic volume when end-systolic pressure changes, and ultimately predicts the left ventricle ability to empty for different afterload values [14].

**Figure 5:**
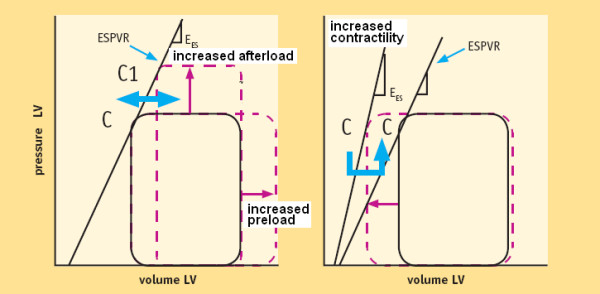
Load changes at constant contractility (left) and contractility changes at constant load (right). Left panel. The graph shows how two additional pressure-volume loops appear with an acute increase in afterload or preload. Contractility is quantified by the ESPVR slope: the Ees (end systolic elastance). Right panel. Increased contractility, is reflected in higher myocardial fiber shortening velocity, with a more highly developed tension peak and a steeper pressure rise, when preload, after load, and heart rate are constant: Ees moves upward and to the left. The left ventricular emptying fraction or ejection fraction (LVEF) is reflected in the ability of the left ventricle to empty. Because myocardial contractility is an important determinant of LVEF, LVEF and contractility are frequently considered to be interchangeable. But they are not the same: thus it is possible to have low LVEF despite normal contractility when LV afterload is excessive. Alternatively, LVEF may be nearly normal despite decreased myocardial contractility if LV afterload is low. (Modified from Little WC. Assessment of normal and abnormal cardiac function. Chapter 15, page 480. In Braunwald Zipes Libby Heart disease, 6th edition, W. B Saunders Company, 2001)

Contractility is quantified by the angular coefficient (or slope) of the ESPVR relation: the Ees (end systolic elastance) (Fig. 5).

## Contractility and heart rate

The heart contractility dependence on increasing heart rates has been established in most mammalians.

**Figure 6:**
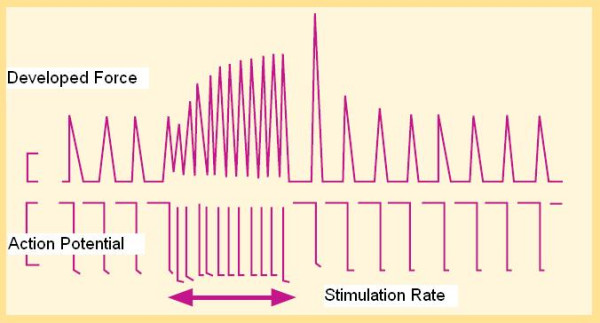
Force-frequency relation or Bowditch treppe. Developed force of contraction in the isolated papillary muscle at increasing stimulation rates. The stimulus rate is shown as the action potential duration on an analog analyzer. The tension developed by papillary muscle contraction is shown as developed force. An increased stimulation rate increases the force of contraction. On cessation of rapid stimulation, the contraction force gradually declines. Heart rate is a leading determinant of cytosol calcium concentration, and strictly linked to contractility. In the healthy heart, a frequency increase up to 180 beats per minute provides for faster systolic calcium SR release (increased contractility or developed force) and for faster diastolic SR calcium reuptake (positive lusitropic effect). (Modified from Opie LH. Normal and abnormal cardiac function. Chapter 14, page 443. In Braunwald Zipes Libby, Heart disease, 6th edition, W. B Saunders Company, 2001).

The inherent ability of ventricular myocardium to increase its strength of contraction independently of neurohormonal control, in response to an increase in contraction frequency is known as frequency treppe [4] (Fig. 6). In humans this myocardial property causes the contractile force to rise, as contraction frequency is increased from 60 to about 180 bpm and to then decline with further increase in frequency (the force-frequency relation "FFR") [6].

### Molecular basis

Heart rate is a leading determinant of cytosol calcium concentration, and strictly linked to the contractility levels. In the healthy heart, a frequency increase up to 180 beats per minute provides systolic faster calcium SR release (increased contractility or developed force) and diastolic faster SR calcium reuptake (positive lusitropic effect).

Up-regulation of Ca^2+ ^entry through Ca^2+ ^channels by high rates of beating (HFIUR of ICa) is involved in the frequency-dependent regulation of contractility: this process is crucial in adaptation to exercise and stress [5]. This regulation is rapid, (the steady state is reached rapidly within few seconds for each heart rate level), intrinsic to the myocardium cell, with no need to be driven from neuronal or hormonal controls (Fig. 1, Fig. 6).

### Cellular and myocardial fiber level

This property has been definitively established in the human heart in experimental settings using cardiomyopathic myocardial strips.

**Figure 7:**
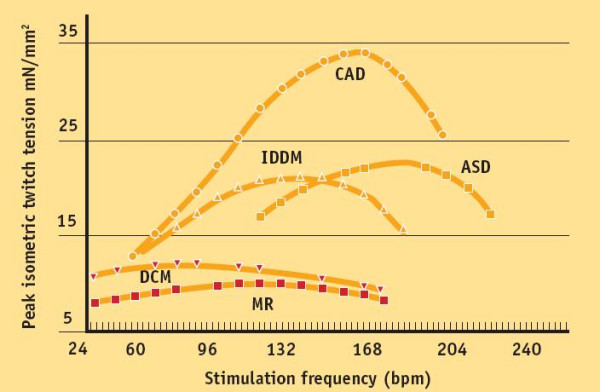
Plots of average steady-state isometric twitch tension versus stimulation frequency in non-failing and failing myocardium. Measurements of twitch tension in isolated left-ventricular strips from explanted cardiomyopathic hearts compared with non-failing hearts show reduction in peak rates of generation and relaxation of twitch tension and a decrease in slope of tension rate vs. contraction frequency The FFR of these failing groups both exhibit a *negative treppe *at contraction frequencies above about 100 bpm. The contraction frequency at which the FFR begins its descending limb ("optimum stimulation frequency") declines progressively in the order: ASD (atrial septal defect), CAD (coronary artery disease), IDDM (diabetic myopathy), MR (mitral regurgitation), DCM (dilated cardiomyopathy). (Modified from: Mulieri AL. In "Heart Metabolism in Failure" R.A. Howarth Ed. 1997. The role of myocardial force-frequency relation in left ventricular function and progression of human heart failure)

Measurements of twitch tension in isolated left-ventricular strips from explanted cardiomyopathic hearts compared with non-failing hearts show reduction in peak rates of generation and relaxation of twitch tension and a decrease in slope of tension rate vs. contraction frequency [15,16] (Fig. 7).

The FFR of these failing groups both exhibit a *negative treppe *at contraction frequencies above about 100 bpm.

Presence of a negative treppe in the working range of heart rates may constitute an additional liability beyond mere depression of the wall tension since this may contribute to an accelerated progression of heart failure. In patients in end stage failure the peak of the FFR occurs at such a low frequency that there is a negative treppe over the entire in vivo range of heart rates. The contraction frequency at which the FFR begins its descending limb ("optimum stimulation frequency") declines progressively in the order: atrial septal defect, coronary artery disease, diabetic myopathy, mitral regurgitation, dilated cardiomyopathy. This suggests that a correlation between severity of myocardial disease and optimum contraction frequency may exist [15,16].

In more severe heart failure the peak of the FFR is shifted sufficiently to lower frequencies so that it has a negative slope over the entire range of in vivo heart rates (i.e., 80–150 bpm). The weakening of contractile strength as heart rate rises suggest the possibility that in vivo, a sudden increase in heart rate could predispose the ventricle to being stretched by venous return.

While the FFR is well known in the physiological lab [5,17,18], with extensive studies in isolated strips of failing myocardium [15,16], in animal models of heart failure [19,20], till now its knowledge and use in the clinical setting is extremely limited [21-26].

### Fetal gene program: back from the future

**Figure 8:**
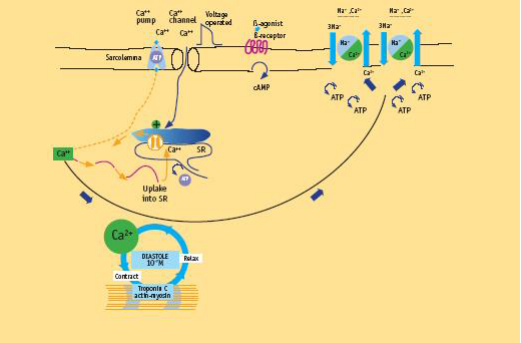
Molecular basis of contractility in failing heart. There is increasing evidence that disturbances in calcium handling play a central role in the disturbed contractile function in myocardial failure. The sarcoplasmic reticulum calcium ATPase (SERCA) is depressed both in function, as well as in expression. At the same time the sarcolemmal sodium-calcium (Na^+^/Ca^2+^) exchanger is increased both in function and in expression. The result is a characteristic change in calcium homeostasis with decreased diastolic uptake of calcium into the sarcoplasmic reticulum with subsequently reduced calcium release during the next systole, resulting in reduced contractile performance. At the same time increased capacity of the sodium-calcium exchanger extrudes intracellular calcium ions to the extra-cellular space, thereby rendering these ions unavailable for the contractile cycle. Intracellular Ca^2+ ^handling is abnormal in heart failure and cause systolic and diastolic dysfunction. The mRNA and protein levels of the Na^+^/Ca^2+ ^exchanger are increased in myocites from heart failure patients and correlates inversely with the SERCA mRNA levels. The augmentation in Na^+^/Ca^2+ ^exchange activity is a compensatory response to the reduction in Ca^2+ ^reuptake caused by a decrease in SERCA2. But enhanced Na^+^/Ca^2+ ^exchange instead of SRCa^2+ ^reuptake is an energy-wasting process: ATP consumption to extrude cytosolic Ca^2+ ^from the myocyte is almost doubled with respect to the normal SRCa^2+ ^reuptake.

"As the ventricle fails, there is a change in the ventricular gene expression pattern from the normal adult pattern to that normally observed only during fetal life. There is a down regulation of the calcium uptake pump (SERCA2) and of the fast-contracting myosin heavy chain. The fetal program may be activated from cytosolic calcium overload, by adding phosphate groups to enzymes that normally inhibit the fetal program [6]" (Fig. 8).

**Figure 9:**
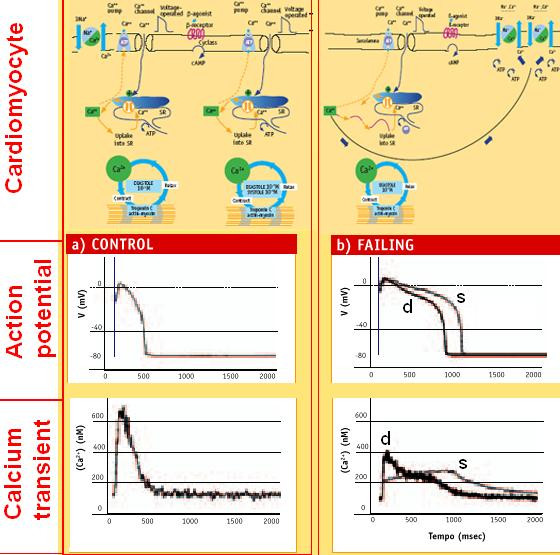
Molecular pathopysiology, action potentials and calcium transients in isolated myocytes of normal (A) vs. failing (B) hearts. Upper panels. Left: normal myocyte. Right: failing heart myocytes show depressed SERCA both in function, and in expression; the sarcolemmal sodium-calcium (Na^+^/Ca^2+^) exchanger is increased both in function and in expression, and correlates inversely with the SERCA levels. Lower panels: action potential and intracellular calcium transient. The action potentials recorded in myocytes isolated from the failing hearts (right) are markedly prolonged compared with that in a myocyte from a normal heart (control, left). The intracellular calcium transients measured with the fluorescent calcium indicator fura-2 are also markedly abnormal in myocytes isolated from the failing heart (right). Compared with a normal myocyte (control, left), the failing myocyte shows (plot s) an attenuated cytosolic Ca^2+ ^rise with depolarization and a markedly delayed return to baseline. The intracellular calcium transient (plot d) from a myocyte with isolated diastolic dysfunction (normal cytolsolic Ca^2+ ^systolic release, delayed cytosolic Ca^2+ ^diastolic removal) shows a normal rise with depolarization and a markedly delayed return to baseline. These abnormalities reflect the altered expression or function of key calcium-handling proteins and contribute to the abnormal action potential in the top illustration. (Modified from: O'Rourke B, Kass DA, Tomaselli GF, et al. Mechanisms of altered excitation-contraction coupling in canine tachycardia-induced heart failure I. Circ Res 1999; 84: 562–70.)

Changes in the calcium cycle are fundamental to the impaired contractile performance of the failing heart. The SR calcium stores are severely depleted because of the combined effects of depressed calcium uptake into the SR resulting from decreased SERCA activity, both down-regulated and inhibited. Thus, the calcium ions entering with depolarization are unable to trigger the release of enough calcium to generate a normal calcium transient (Fig. 9). There is a close relationship between the depression of SERCA in human heart failure and the depressed force-frequency relationship. Paradoxically, the diastolic calcium level is higher than normal. Starting from this higher level, as the heart rate increases, the calcium ions enter more rapidly through the calcium channels than can be extruded through the Na^+^/Ca^2+ ^exchange, so that the diastolic levels rise, as does the diastolic tension.

Muscle strips prepared from patients with severe heart failure behave very differently from normal muscle, in that there is hardly any response to an increased stimulation frequency. Whereas in strips from normal hearts, optimal force development is reached at rates of about 150 to 180 beats/min, in patients with cardiomyopathy an increased heart rate produces a decreased twitch tension (Fig. 7). In addition, the diastolic tension rises markedly with the stimulation frequency, compatible with a rate-induced cytosolic calcium overload causing diastolic dysfunction [6].

### Present limits of end systolic elastance (ESPVR slope) for contractility measurement

This standard, historically accepted, rest-assessed contractility, is limited because it is an invasive index, but especially because it fails to take into account frequency-dependent regulation of contractility: ultimately developed in the evolutional scale, as a typical feature of more advanced mammalian species, absent in fetal life and in adults with heart failure-induced regression of the contractile mechanism, the frequency-dependent control of transmembrane Ca^2+ ^entry via voltage-gated Ca^2+ ^channels provides mammalian cardiac cells with a highly sophisticated short-term system for regulation of intracellular Ca^2+ ^homeostasis.

The impossibility of separating the cellular mechanism of contractility changes from those of load or heart rate is now clear. "Thus, there is a clear overlap between contractility, which should be independent of load or heart rate, and the effects of load and heart rate on the cellular mechanism. Hence, the traditional separation of inotropic state from load or heart rate effects as two independent regulators of cardiac muscle performance is no longer simple now that the underlying cellular mechanisms have been uncovered [6]." This topic is not important only as a speculative concept, but especially clinically: in fact as the heart fails, there is a change in the ventricular gene expression pattern from the normal adult pattern to that normally observed only during fetal life, as a memory of primordial contraction patterns, with an inversion of the normal positive slope of the relation: the systolic calcium release and diastolic calcium reuptake process is lowered at the basal state and, instead of accelerating for increasing heart rates, it slows down. Since the assessment of FFR shows initial alteration of contractility, as an intermediate step between normal and abnormal contractility at rest, a practical index to measure it is mandatory.

**Figure 10:**
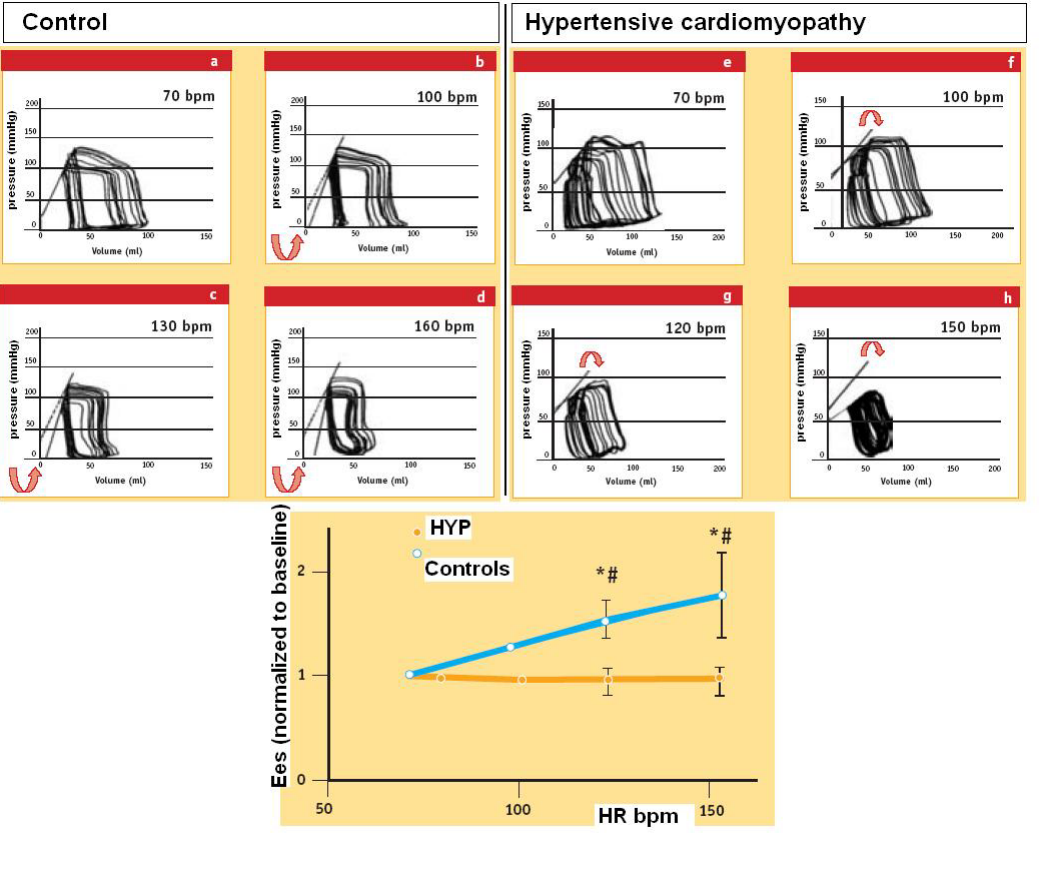
Force-frequency relationship in the cath lab. During a pressure-volume loop study the contractility was quantified at baseline and during heart rate increase (atrial pacing). At each incremental heart rate the upper left corners of the loops define the LV end-systolic pressure-volume relation (ESPVR). The slope of the ESPVR is the end-systolic elastance (Ees). Upper left panel. During atrial pacing in a control subject (Control) for higher heart rates the ESPVR is shifted leftward, and Ees increases: contractility increases as heart rate increases. Upper right panel. A patient with severe LV hypertrophy (Hypertensive cardiomyopathy) displays a decrease in the ESPVR slope for heart rate increases: from 70 to 100 bpm and at further increases in heart rate (from 100 to 120 and to 150 bpm): contractility decreases at higher heart rates. Lower panel. For each study group end-systolic elastance (Ees, mean value ± SD) is plotted at different heart rates during rapid atrial pacing; for the 8 control (controls, non-LVH) patients, the Ees increased with each increment in heart rate. In contrast, Ees fell at faster rates in hypertensive (HYP) subjects. (Modified from: Liu C. Diminished contractile response to increased heart rate in intact human left ventricular hypertrophy. Circulation 1993; 88:1893)

Since end-systolic elastance (Ees), expressing the slope of the in-vivo, end-systolic ventricular pressure vs chamber volume relation, is the most "foolproof' window into in vivo myocardial contractility, Ees should be measured at each heart rate step increase, as made by Liu and coworkers [21] (Fig. 10).

But measuring Ees for increasing heart rates is impractical: increasing heart rates obtained with temporarily pacing has to be adjunct to the LV conductance catheter, the LV pressure catheter, the vena cava balloon, and to afterload changes. Proof of this is that only Liu [21] adopted this method in humans. (Table 1).

**Table 1:** Force-frequency relationship from the experimental lab to clinical applications

**Author**	Feldman	Bhargava	Hasenfusss	Liu	Inagaki	Schuler	Dehmer	Lavie
**Journal**	J Clin Invest	Am J Cardiol	Eur Heart J	Circ	Circ	Am j Cardiol	Am J Cardiol	Chest
**Year**	1988	1988	1994	1993	1999	1982	1981	1989
Method	**cath lab**	**cath lab**	**cath lab**	**cath lab**	**cath lab**	**nuc**	**nuc**	**nuc**
**FORCE**	SP/ESV	dP/dt	dP/dt	Ees	dP/dt	SP/ESV	SP/ESV	SP/ESV
**TREPPE**	Yes	Yes	Yes	Yes	Yes	Base- peak	Base-peak	Base-peak
**HR increase**	PM	PM	PM	PM	PM EX ISO	EX	EX	EX
**PTS# Disease**	DC 7	DC 5	DC 9	HYP 10	HYP 17	AR 14	AR 17	MR 11
**FFR Upsloping**	3	-	-	-	7	7	11	7
**Flat-Biph**	-	2	-	10	10	7	2	2
**Neg**	4	3	9	-	-	-	4	2
**Control #**	6	3	8	8	10	9	15	-
**FFR Upsloping**	6	3	8	8	10	9	15	-
**Flat-Biph**	-	-	-	-	-	-	-	-
**Neg**	-	-	-	-	-	-	-	-

PM = atrial pacing; EX = exercise; ISO = isoproterenol ; DC = dilated cardiomyopathy; HYP = hypertensive cardiomyopathy; CHD = coronary artery disease; AR = aortic regurgitation; MR = mitral regurgitation; FFR = force-frequency relation

Several attempts have been made to transfer the force-frequency relationship from the experimental lab to clinical applications. Such attempts have been based on invasive evaluation in cath lab (Feldman 1988, Bhargava 1988, Hasenfuss 1994, Liu 1993, Inagaki 1999), or noninvasive evaluation with radionuclide scintigraphy (Schuler 1982, Dehmer 1981, Lavie 1989). The extensively adopted maximum rate of pressure rise (max dP/dt) for force measurement is largely preload and afterload dependent. Since End-systolic elastance (Ees), is almost insensitive to changes in preload and afterload, Ees should be measured at each heart rate step increase, as done by Liu and coworkers.

But measuring Ees for increasing heart rates is impractical: increasing heart rates obtained with atrial pacing has to be adjunct to the LV conductance catheter, the LV pressure catheter, the vena cava balloon, and the afterload changes. Proof of this is that only Liu adopted this method in humans.

The scintigraphic approach is noninvasive, but requires exposure to ionizing radiations and – due to limited temporal resolution – allows the measurement of SP/ESV only at baseline at peak exercise. The pattern of the force-frequency relationship over a spectrum of different heart rates cannot be assessed.

If assessment of Ees is difficult under clinical conditions at fixed heart rates, assessment of Ees for increasing heart rates is much more difficult.

### The Suga index (SP/ESV ratio) for increasing heart rates: the link toward the stress echo lab FFR measurement in a practical clinical method

**Figure 11:**
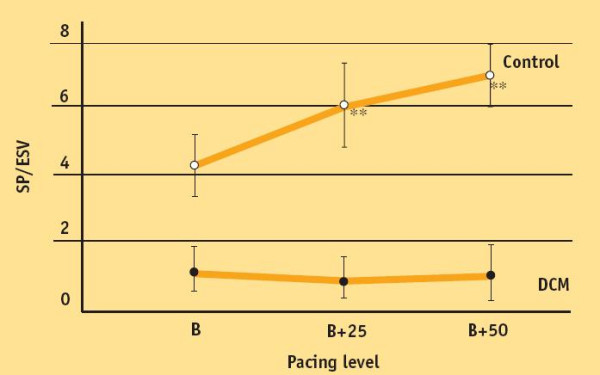
The Suga (SP/ESV) index instead of end-systolic elastance for FFR measurement. Since End-systolic elastance (Ees), expressing the slope of the in-vivo, end-systolic ventricular pressure vs. chamber volume relation, is the most "foolproof' window into in vivo myocardial contractility, Ees should be measured at each heart rate step increase. A simpler approach was utilized by Feldman and co-workers by measuring SP/ESV ratio at baseline, and for pacing induced heart rate increase to 25 and 50 bpm beyond basal heart rate. *Feldman *showed that 7 patients with dilated cardiomyopathy (DCM) demonstrated little or no significant enhancement in SP/ESV ratio during atrial pacing tachycardia. The lack of improvement in cardiomyopathy patients has been contrasted to patients with normal ventricular function (Control) who demonstrated significant increase in SP/ESV ratio. SP/ESV ratio is simpler than Ees measurement, and equally provides knowledge of up-sloping vs flat-biphasic force-frequency relationship. (Modified from: Feldman MD, Alderman JD, Aroesty JM, Royal HD, Ferguson JJ, Owen RM, et al. Depression of systolic and diastolic myocardial reserve during atrial pacing tachycardia in patients with dilated cardiomyopathy. *J Clin Invest *1988; 11:1661–9)

A simpler approach was utilized by Feldman and co-workers [26] in DCM pts vs. normal hearts, by measuring the SUGA index (SP/ESV ratio, instead of Ees) at baseline, and for pacing induced heart rate increase to 25 and 50 bpm beyond basal heart rate. SP/ESV ratio measurement is simpler than Ees measurement, and equally provides knowledge of an up-sloping, flat, or biphasic Bowditch treppe (Fig. 11).

## Force-frequency relationship in the stress echo lab: a practical, noninvasive, modern approach to contractility

**Figure 12:**
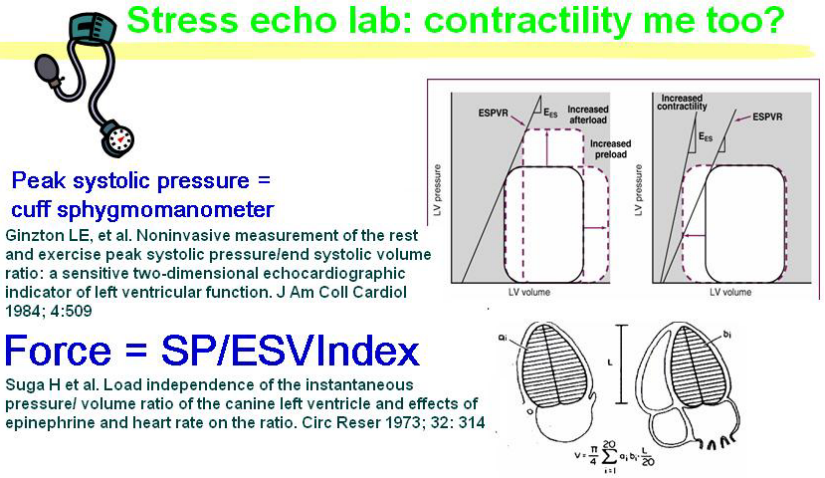
Stress echo lab: contractility me too? *Blood pressure analysis*. One investigator records all blood pressures at rest and during exercise during the study. The blood pressure recording is made using a manometer sphygmomanometer and the diaphragm of a standard stethoscope. *Echocardiography *is performed using conventional two-dimensional echocardiography and tissue harmonic imaging and digitized on-line into a quad screen, cineloop format. Left ventricular end-systolic volumes are measured from apical four and two chamber view, using the biplane discs-method. To build the force-frequency relationship, the force is determined at each step as the ratio of the systolic pressure (cuff sphygmomanometer)/end-systolic volume index (biplane Simpson rule/body surface area).

Non-invasive methods [27-30] have been proposed to assess the rest-peak stress change in inotropic state, based upon the assumption that positive inotropic interventions are mirrored by smaller end-systolic volumes and higher end-systolic pressures (Table 1). During bicycle stress echocardiography, dobutamine or pacing stress, continuous 2D echo monitoring is performed by protocol and blood pressure, ECG and left ventricular volumes are obtained at each step, providing the basic information required to build a force-frequency relationship over a wide range of frequencies (Fig. 12). A totally noninvasive estimation of force-frequency relation during stress in the echo lab is theoretically appealing for the identification of limited contractile reserve and latent global left ventricular dysfunction.

This method is similar to the previously proposed ones but is totally noninvasive, with echocardiography used to assess LV volumes during exercise and cuff blood pressure to estimate peak systolic pressure as an index of end-systolic pressure [31-33].

### Bowditch treppe and stress echo. Methodology

During (exercise, DOB or pacing) stress echocardiography continuous 2D echo monitoring is performed by protocol and blood pressure, ECG and left ventricular volumes are obtained at each step, providing the basic information required to build a force-frequency relation over a wide range of frequencies [34].

**Figure 13:**
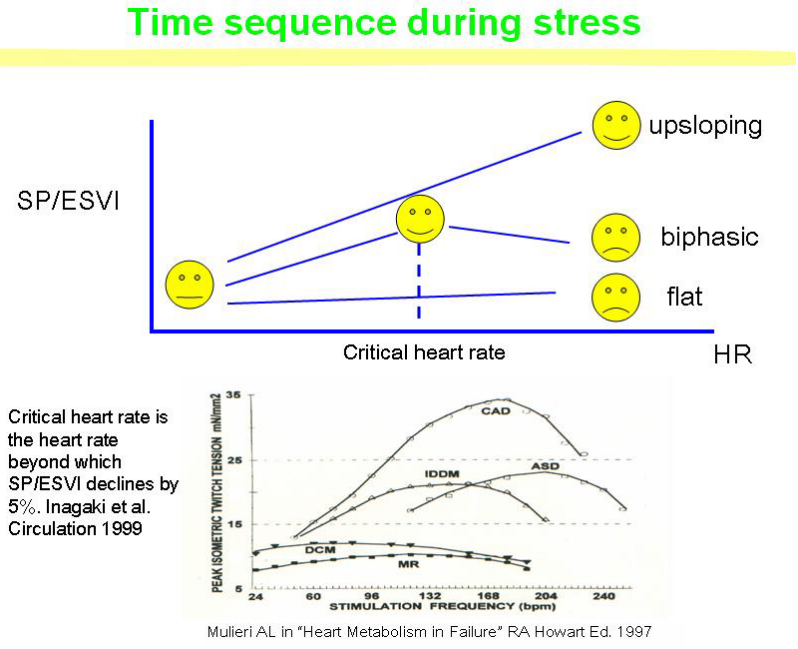
FFR, from myocardial strips to the echo lab. Time sequence during stress echo (upper panel). The force frequency relation is built off line. The force-frequency relationship is defined up-sloping when the peak exercise SP/ESV index is higher than baseline and intermediate stress values; biphasic, with an initial up-sloping followed by a later down-sloping trend, when the peak exercise systolic pressure/end-systolic volume index is lower than intermediate stress values; flat or negative, when the peak exercise systolic pressure/end-systolic volume index is equal to or lower than baseline stress values. The critical heart rate (or optimum stimulation frequency) is defined as the heart rate at which systolic pressure/end-systolic volume index reaches the maximum value during progressive increase in heart rate; in biphasic pattern, the critical heart rate is the heart rate beyond which the systolic pressure/end-systolic volume index has declined by 5%; in negative pattern the critical heart rate is the starting heart rate. The critical heart rate (or optimum stimulation frequency) is the human counterpart of the treppe phenomenon in isolated myocardial strips; the optimal heart rate is not only the rate that would give maximal mechanical performance of an isolated muscle twitch, but also is determined by the need for diastolic filling. ASD = atrial septal defect; CAD = coronary artery disease; IDDM = diabetic myopathy; MR = mitral regurgitation; DCM = dilated cardiomyopathy.

This approach is based on serial assessment of these variables at different exercise steps so that the force-frequency pattern (up sloping, flat, and biphasic) can be assessed (Fig 13).

#### Baseline and stress echocardiography

The patient undergoes transthoracic echocardiography at baseline and at each 10 beat frequency increase during stress. This is performed using conventional two-dimensional echocardiography and tissue harmonic imaging, and digitized on-line into a quad screen, cineloop format. Images are also recorded on half-inch S-VHS videotape. Left ventricular end-diastolic and end-systolic volumes are measured from apical four and two chamber view, by an experienced observer using the biplane discs-method [35,36] (Fig. 12). Only representative cycles are measured and the average of three measurements is taken. The endocardial border is traced, excluding the papillary muscles. The frame captured at the R wave of the ECG is considered to be the end diastolic frame, and the frame with the smallest left ventricular cavity the end systolic frame.

#### Blood pressure analysis

One investigator records all blood pressures at rest and during exercise during the study. The blood pressure recording is made using a manometer sphygmomanometer and the diaphragm of a standard stethoscope (Fig. 12).

#### End-systolic pressure-volume determination

**Figure 14:**
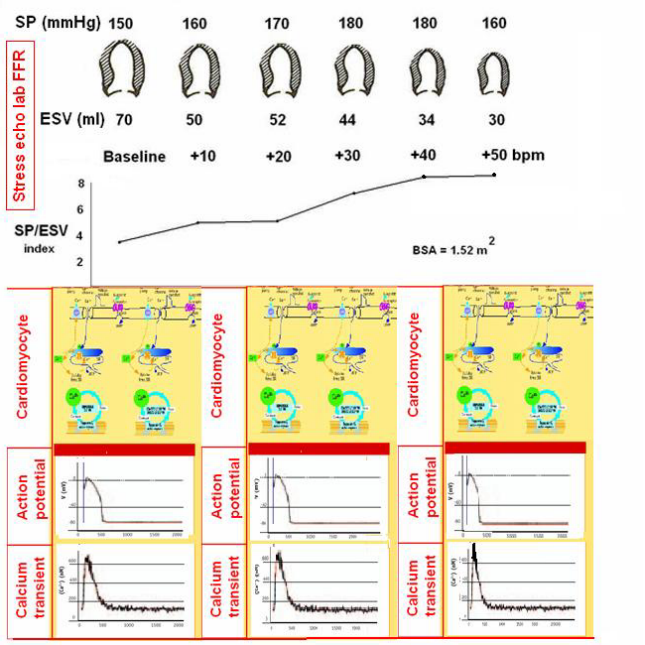
Force-frequency curve with stress echo in a normal subject. Upper panel: On the left, systolic blood pressure by cuff sphygmomanometer (SP, first row); left ventricular end-systolic volumes calculated with biplane Simpson method (ESV, second row); heart rate increase during stress (bpm, third row); in the lowest row, the force-frequency curve built off-line with the values recorded at baseline (second column), and at different steps (third, fourth, fifth column) up to peak stress (sixth column). An increased heart rate is accompanied by an increased systolic pressure with smaller end-systolic volumes (normal up sloping force-frequency relation). Lower panel: molecular basis (first row), action potential (second row) and calcium transient (third row) of myocytes at baseline (first column), intermediate stress (second column) and peak stress (third column). In the normal heart increase in heart rate is accompanied by an increase in myocardial contractile performance (up-sloping FFR). At higher heart rates more and faster "cascade" calcium is released from the SR: more calcium is available in the cytoplasm for C troponin interaction and contraction. Equally calcium reuptake is more and faster in diastole. Both action potential and calcium transient are rapidly peaking in systole at each stress step.

**Figure 15:**
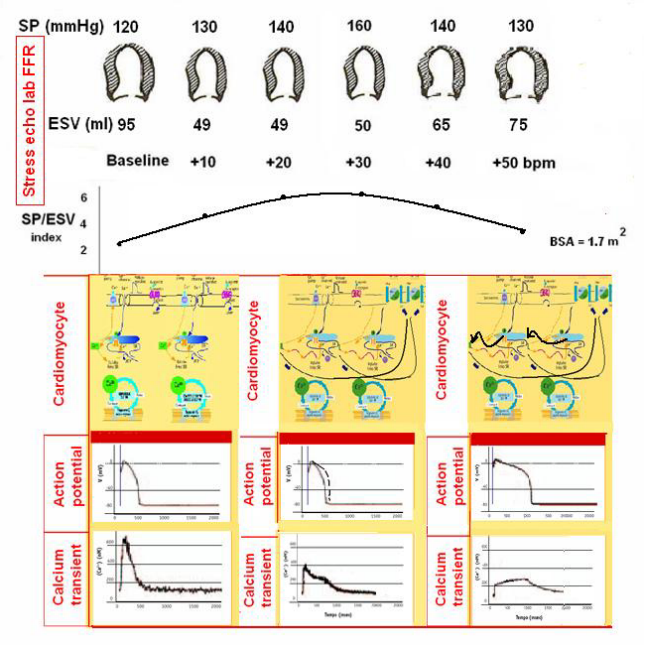
Force-frequency curve with stress echo in a subject with latent LV dysfunction without dilation. Upper panel. On the left, systolic blood pressure by cuff sphygmomanometer (SP, first row); left ventricular end-systolic volumes calculated with biplane Simpson method (ESV, second row); heart rate increase during stress (bpm, third row); in the lowest row, the force-frequency curve built off-line with the values recorded at baseline (second column), and at different steps (third, fourth, fifth column) up to peak stress (sixth column). The force-frequency relation is biphasic, with an initial up-sloping trend followed by a later down-sloping trend. Lower panel: hypothetical molecular basis (first row), action potential (second row) and calcium transient (third row) of myocytes at baseline (first column), intermediate stress (second column) and peak stress (third column). In latent failing myocytes calcium transient can be normal at baseline, but abnormal at higher heart contraction rates: compared with a normal baseline pattern (first column), at intermediate stress (second column) delayed cytosolic Ca^2+ ^diastolic removal occurs; further dysfunction (cytolsolic Ca^2+ ^attenuated rise with depolarization and a markedly delayed return to baseline) occurs at higher heart rates. When the heart beats at frequencies beyond the CHR, when calcium is extruded from the myocyte instead of re-entry in the SR, the O2 consumption for each unit of force developed is doubled; the combination of decreased cardiac force development and increased oxygen uptake indicates decreased efficiency of cardiac work.

**Figure 16:**
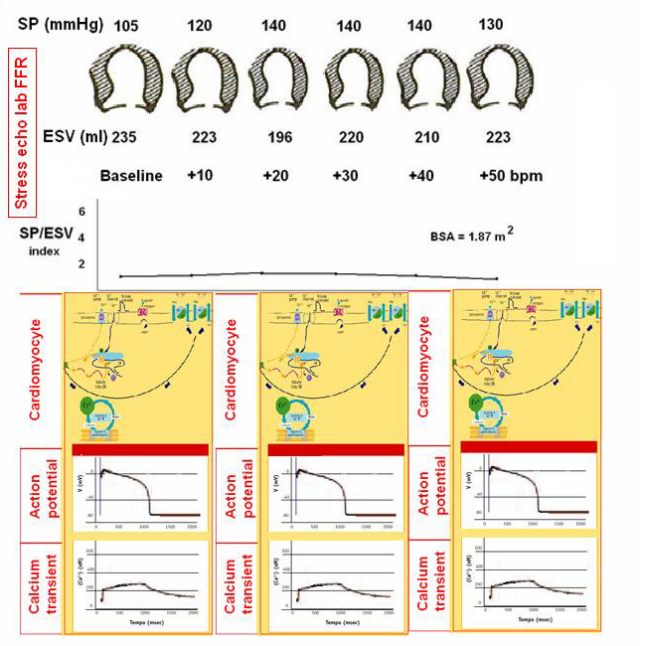
Force-frequency curve with stress echo in a subject with dilated cardiomyopathy and depressed baseline left ventricular function (EF% = 30%). On the left: systolic blood pressure by cuff sphygmomanometer (SP, first row); left ventricular end-systolic volumes calculated with biplane Simpson method (ESV, second row); heart rate increase during stress (bpm, third row); in the lowest row, the force-frequency curve built off-line with the values recorded at baseline (second column), and at different steps (third, fourth, fifth column) up to peak stress (sixth column). An increased heart rate at peak exercise is accompanied by no changes in end-systolic volumes (abnormal flat force-frequency relation). Lower panel: molecular basis (first row), action potential (second row) and calcium transient (third row) of myocytes at baseline (first column), intermediate stress (second column) and peak stress (third column). The action potentials are markedly prolonged at baseline and during stress in patients with advanced heart failure; calcium cycling is slow at basal heart rates and even more at higher heart rates. These abnormal patterns are related to a profound derangement of the contractile machinery in the failing myocyte: fewer calcium membrane channels, fewer RNA levels encoding contractile proteins, fewer and dysfunctioning SERCA. A critical alteration of force-frequency relationship occurs, with an inversion of the normal positive to a flat or negative slope.

To build the force-frequency relationship, the force is determined at each step as the ratio of the systolic pressure (cuff sphygmomanometer)/end-systolic volume index (biplane Simpson rule/body surface area). The force frequency relation is built off line (Fig. 13). The slope of the relationship is calculated as the ratio between SP/ESV (Systolic Pressure/End-Systolic Volume) index increase (from baseline to peak exercise)/heart rate increase (from baseline to peak exercise). The force-frequency relationship is defined up-sloping when peak exercise SP/ESV index is higher than baseline and intermediate stress values (Fig. 14); biphasic, with an initial up-sloping followed by a later down-sloping trend, when peak exercise systolic pressure/end-systolic volume index is lower than intermediate stress values [6,25] (Fig. 15); flat or negative, when peak exercise systolic pressure/end-systolic volume index is equal to or lower than baseline stress values (Fig. 16). The critical heart rate (or optimum stimulation frequency) is defined as the heart rate at which systolic pressure/end-systolic volume index reaches the maximum value during progressive increase in heart rate; in biphasic pattern, the critical heart rate is the heart rate beyond which systolic pressure/end-systolic volume index has declined by 5%; in a negative pattern the critical heart rate is the starting heart rate [25].

The critical heart rate (or optimum stimulation frequency) is the human counterpart of the treppe phenomenon; "in situ, the optimal heart rate is not only the rate that would give maximal mechanical performance of an isolated muscle twitch, but also is determined by the need for diastolic filling [6]" (Fig. 13)

## Conclusion

This proposed approach allows the assessment of a theoretically robust and sophisticated index of left ventricular contractility with an absolute minimum extra-burden of data acquisition and analysis, since all the basic parameters (heart rate, blood pressure and left ventricular volumes) are routinely acquired during exercise stress echo testing [34]. The extra measurements consist of serial evaluation of ventricular volumes and linear interpolation of the force-frequency relationship. This approach is simple, not time-consuming, and highly feasible [31-33]. This index of global contractility is theoretically appealing for the identification of limited contractile reserve and latent global left ventricular dysfunction.

These are all prerequisites for a larger scale testing in the clinical subsets in which the contractility information can be more important – such as patients with latent ventricular dysfunction [37] or advanced chronic heart failure [33].

Noninvasive measurement of pressure/volume relation (the Suga index) [11,26,31] for increasing heart rates during stress in the echo lab could be the practical answer to this new clinical demand in recent years of a dramatic increase in the number of heart failure patients.
